# RHSOFS: Feature Selection Using the Rock Hyrax Swarm Optimization Algorithm for Credit Card Fraud Detection System

**DOI:** 10.3390/s22239321

**Published:** 2022-11-30

**Authors:** Bharat Kumar Padhi, Sujata Chakravarty, Bighnaraj Naik, Radha Mohan Pattanayak, Himansu Das

**Affiliations:** 1 Department of Computer Science & Engineering, Centurion University of Technology & Management, Bhubaneswar 761211, Odisha, India; 2 Department of Computer Application, VSSUT, Burla 768018, Odisha, India; 3 School of Computer Science & Engineering, VIT-AP University, Amravati 522237, Andhra Pradesh, India; 4 School of Computer Engineering, KIIT Deemed to be University, Bhubaneswar 751024, Odisha, India

**Keywords:** credit card fraud risk, feature selection, classification algorithm, rock hyrax algorithm

## Abstract

In recent years, detecting credit card fraud transactions has been a difficult task due to the high dimensions and imbalanced datasets. Selecting a subset of important features from a high-dimensional dataset has proven to be the most prominent approach for solving high-dimensional dataset issues, and the selection of features is critical for improving classification performance, such as the fraud transaction identification process. To contribute to the field, this paper proposes a novel feature selection (FS) approach based on a metaheuristic algorithm called Rock Hyrax Swarm Optimization Feature Selection (RHSOFS), inspired by the actions of rock hyrax swarms in nature, and implements supervised machine learning techniques to improve credit card fraud transaction identification approaches. This approach is used to select a subset of optimal relevant features from a high-dimensional dataset. In a comparative efficiency analysis, RHSOFS is compared with Differential Evolutionary Feature Selection (DEFS), Genetic Algorithm Feature Selection (GAFS), Particle Swarm Optimization Feature Selection (PSOFS), and Ant Colony Optimization Feature Selection (ACOFS) in a comparative efficiency analysis. The proposed RHSOFS outperforms existing approaches, such as DEFS, GAFS, PSOFS, and ACOFS, according to the experimental results. Various statistical tests have been used to validate the statistical significance of the proposed model.

## 1. Introduction

Feature selection (also known as variable selection) is an important topic in the field of data mining. It is motivated by the requirement to choose the “optimal” selection of variables for prediction. The purpose of FS is to find the “best” subsets of features (or variables) for statistical analysis or to build a machine learning model [1,2]. Preprocessing is frequently required in FS to help the classification, prediction, or clustering stages better distinguish or represent the data and different approaches to be followed for feature selection [3,4]. In data mining and machine learning applications, FS is a critical activity that eliminates unnecessary and redundant characteristics and improves learning performance [5,6]. FS decreases dimensionality, removes irrelevant input, improves learning accuracy, and improves result comprehension as a preprocessing step for machine learning [7]. The difficulty for a learning algorithm is focusing its attention on a subset of features while ignoring the rest of the problem. As a result, processing and analyzing such large amounts of data is quite difficult. Without the use of an automated system, extracting valuable information from enormous amounts of data is a difficult task. FS is essential for detecting credit card fraud in large, multi-dimensional [8], and imbalanced datasets [9]. Many optimization algorithms have been used in the past decades to solve the FS algorithm by creating a subset of important features from dimensional datasets [10,11,12,13,14,15,16,17,18,19,20,21,22].

**Figure 1:**
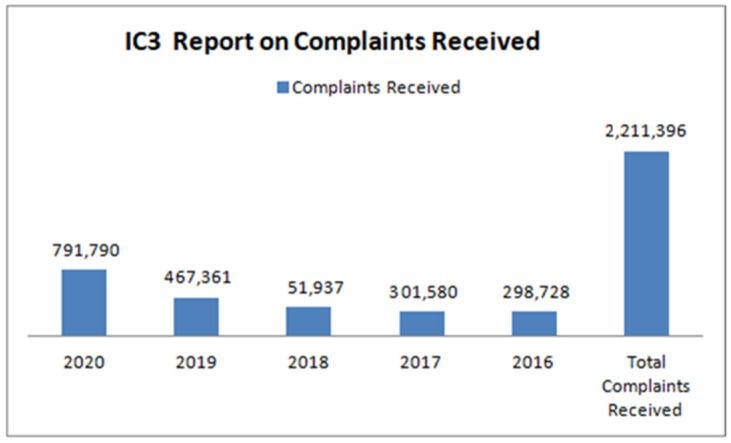
IC3 Report on complaints received.

Filtering and wrapping are two types of FS approaches. If the FS technique is unaffected by the learning algorithm, then it is referred to as a filter approach; otherwise, it is referred to as a wrapper approach. The filter method is faster than the wrapper method in terms of processing time. On the other hand, the filter technique has the major disadvantage of being susceptible to inductive biases in the learning algorithms used to build the classifier. The wrapper approach has a higher processing overhead because it uses learning algorithms to evaluate a subset of features. However, in terms of accuracy, the wrapper strategy may outperform the filter method [1,2,10,11]. A preprocessing step is used in the filter technique [1,3,11] to select the best features. The filter approach’s fundamental flaw is that it completely disregards the impact of the selected feature subset on the induction algorithm’s performance. The wrapper methodology [1,3,11] introduced by Kohavi and John in 1997 is a simple and effective method for dealing with the problem of variable selection. The feature subset selection algorithm is used to wrap around the induction process in the wrapper approach. As part of the function evaluating feature subsets, the feature subset selection algorithm searches for a good subset using the induction process. The wrapper approach works on the simple principle of treating the induction process as a black box. The feature subset selection uses the induction technique as a black box in the wrapper approach, as shown in Figure 1. (i.e., no knowledge of the algorithm is needed, just the interface). The feature subset selection algorithm employs the induction algorithm as part of the evaluation function to conduct a search for a good subset. The wrapper-based FS technique is used in this article for optimal FS. For each individual combination of features, FS employs a search strategy to identify the best-suited features. The number of features increases exponentially with the number of viable solutions in the classic search approach [1,3,10,11].

Grasshopper Optimization [12], Differential Evolution algorithm (DE) [17], Genetic Algorithm (GA) [18], Particle Swarm Optimization (PSO) [19], and Ant Colony Optimization (ACO) [20,21] have all been used to solve the FS problem using wrapper-based FS methods. The disadvantage of this FS method is that it necessitates the tuning of various parameters for better performance. In this context, this paper proposes a new wrapper-based FS approach based on the Rock Hyrax Optimization (RHO) algorithm [23], which can detect credit card fraud [24,25,26,27,28,29,30] in massive and high-dimensional datasets and is considered very important for improving classification performance and fraud detection processes. This method also identifies FS models with small abnormalities in large datasets [31,32] with high precision and focuses on low computation that does not require extensive model-specific parameter settings. It finds the most necessary and pertinent features by upgrading the worst features using the RHO algorithm’s strength. These important characteristics are appropriate for developing a supervised machine-learning model for categorization.

**Figure 2:**
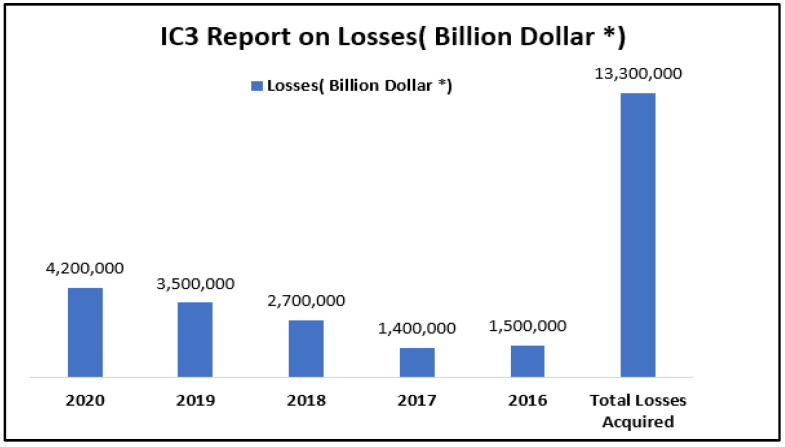
IC3 Report on loss.

Table 1 demonstrates that the Internet Crime Complaint Center (IC3) was formed in May 2000 to process complaints concerning internet crime as defined by the Certified Fraud Examiner Association (CFEA) [33]. Figure 1 and Figure 2 below display aggregate complaints and loss data annually from 2016 to 2020. During that time, IC3 received a total of 2,211,396 complaints and USD 13.3 billion in revenue losses.

The main contributions of this study are that it presents a novel FS method based on the Rock Hyrax Swarm Optimization algorithm, as well as a detailed experimental comparison of numerous FS approaches such as DEFS, GAFS, PSOFS, ACOFS, and RHSOFS. Thus, it also presents optimum features for creating an effective credit card fraud detection system. 

Several performance measures have been applied to FS approaches, and performance evaluation was carried out using extensive experiments on credit card datasets using classification algorithms such as NB, SVM [21,25,32], KNN, and DT, and the results were compared to show that the experimental data were significant. The key consequences of this presented technique are that it reduces the overfitting concerns that arise when datasets are imbalanced [27] and increases the model’s generalizability. Intrusion detection, spam mail detection systems, important medical disease classification, sophisticated picture classification, and industrial automation systems are examples of applications that require large and complex data processing.

The remaining sections of the paper are organized as follows. Section 2 discusses the related work on feature selection algorithms and their impact on application research areas. Section 3 briefly discusses feature subset selection modeling and key problem formulation. Section 4 thoroughly describes the proposed RHOFS methodology. Section 5 presents experimental and statistical result analysis of various FS methods, as well as key issue discussion, and Section 6 concludes with futile suggestions and future work. 

## 2. Literature Review

In the paper [1], the relationship between optimal feature subset selection and relevance was investigated, as well as the wrapper method to feature subset selection utilizing naïve Bayes and decision trees. This paper [2] discusses the fundamental difficulties in FS, such as feature relevance, redundancy, the characteristics and performance of different FS methods, and how to choose the best method for a given application. In the paper [3], the proper definitions of the objective function, as well as feature creation, were explored. It also looked at feature ranking and multivariate FS, as well as efficient search algorithms and ways for determining feature validity. The paper [4] focused on various typical methods of FS and extraction, as well as comparisons of each method. The paper [5] provided a detailed assessment of semi-supervised FS strategies, outlining the benefits and drawbacks of each method. The paper [7] focused on the filer model and created a new FS method that can successfully remove both unnecessary and redundant features while being less computationally expensive than existing algorithms. The paper [9] provided a wrapper-based FS strategy for selecting the most relevant features based on the artificial electric field optimization algorithm. A new method for selecting features in biomedical data is proposed in the paper [11]. A novel FS technique based on the real-valued grasshopper optimization algorithm was proposed in this paper [12]. The jaya optimization algorithm [15] has been used to construct a unique and prominent wrapper-based FS model with an emphasis on a low-computing FS model that does not require sophisticated algorithm-specific parameter tuning. The goal of the paper [16] was to find a way to reduce the time complexity of wrapper-based FSS with an embedded K-Nearest-Neighbor (KNN) classifier by building a classifier distance matrix and incrementally updating it to speed up the calculation of relevance criteria in evaluating the quality of candidate features. The paper [17] proposed a Differential Evolution (DE) optimization technique for FS, and the result of DE was compared with GA and PSO. The use of genetic algorithms to solve the feature subset selection problem using neural network classifiers was presented in the paper [18]. The implementation of FS in intrusion detection in wireless sensor networks is provided in the paper [19], which is based on PSO and PCA space. The outcome of the approach is compared to that of GA. The Ant Colony Optimization technique for FS is provided in the paper [20,21]. The paper [23] suggested a new swarm intelligence technique based on the behavior of rock hyrax swarms. The proposed algorithm can also balance the exploration and exploitation phases, making it suitable for a wide range of optimization problems. In the paper [24], an improved Credit Card Risk Identification (CCRI) technique for detecting fraud risk is described, which is based on feature selection algorithms such as Random Forest and Support Vector Machine (SVM) classifier. The paper [25] describes an SVM-type FS strategy that uses artificial variables and mutual information to filter out noisy variables from high-dimensional metabolome data. In the paper [26], they presented a credit card fraud detection model and the necessity of using a feature selection approach. Some of the most prominent supervised and unsupervised machine learning algorithms were used to detect credit card thefts in a severely skewed dataset [30]. The analysis and comparison studies of different machine learning algorithms and boosting machine learning algorithms for fraud detection are discussed in papers [31,32].

## 3. Problem Definition

This section describes the problem formulation applied in this paper. To increase the performance of classification models, FS refers to a method for selecting an optimum subset of input features from the entire dataset. It simply picks out the elements that matter in the decision-making process. To lower the computing cost of the problem, it generates and selects the most effective subset of features by removing redundant and irrelevant features. This is an NP-hard problem [13], meaning it cannot be solved in polynomial time. The goal is to obtain the best subset of features to increase the classification process’ performance. The following are the four steps that make up the best FS: (i) a subset of features is generated; (ii) using these subsets of characteristics, evaluate and compare fitness levels; (iii) verify that the termination conditions have been met, if not, repeat steps (i) and (ii); validate the results using the best subset of characteristics.

The problem formulation for FS is performed by selecting *d* important features from a set of *D* features, which is represented in Equation (1), shown below:

(1) f(x)=min_err(d) and d⊂D

$\mathrm{Minimize}\;f\left(x\right)$*,*  Subject to Condition, x=|D| and x≥0.

Using the optimized subset features, Equation (1) reduces error in each iteration stage, thus increasing classification accuracy in the proposed model. 

## 4. Proposed Model

RHSO (Rock Hyrax Swarm Optimization) is a meta-heuristic based on the natural behavior of rock hyrax swarms. The RHSO algorithm simulates the collective behavior of rock hyraxes to find food and their unique way of looking at it. Rock hyraxes live in colonies or groups, with a dominant male keeping a close eye on the colony to ensure its protection. The algorithm seeks out the best solutions by incorporating both local heuristics and prior knowledge into the construction of the best subset of features to improve the classification process’ performance [23].

**Figure 3:**
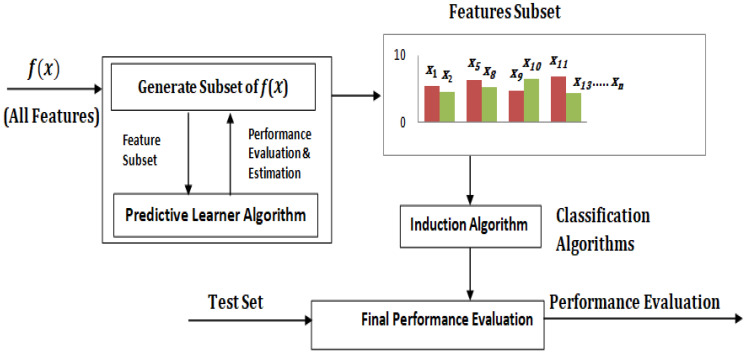
Feature Selection Models Block Diagram.

The RHSOFS detailed functioning model is depicted in Figure 3, which separates the whole dataset into training and testing sets. The training data are entered into the optimization technique. (i.e., *f*(*x*)) to find the best suitable optimum features. The classification algorithm is fed the optimum subset of features (i.e., *f*(*x*)), train, and test data to evaluate the model’s performance. Equation (1) can be used to represent the selection of the most optimal features. Equation (2) reduces the error in each iteration using the specified characteristics, increasing the classification accuracy in the process.

Population size, generation number, initial weighting factors, cognitive and social scaling factors, and probabilities of mutation and crossover are some of the regulatory parameters for population-based algorithms. To achieve the best results, these parameters must be fine-tuned, and the performance of optimization methods is determined by parameter fine-tuning; otherwise, these parameter values may end up in the optimization algorithm in a local optimum stage, increasing the computational cost of the optimization algorithm problem. To address the aforementioned concerns, the RHOSFS technique is applied. This approach is applied to create the best subset of input features to increase the efficiency of the classification process. The RHSOFS comprehensive functional model is depicted in Figure 3. Below is a detailed description of the proposed RHOSFS approach.

First, generate, select, and examine a random sample of a binary (0, 1) population for the total number of input features for FS. Create a feature subset that is equal to 1 for each representation of the input population. For the computation of fitness, the extracted optimal input features are fed into classification models such as NB, SVM, KNN, and DT to compute fitness. The goal of this research work is to find the best subset of input features that reduce the model’s fitness while also improving its accuracy.

(2) $$err\left(x_{i}\;\right)=actual\_output\;\left(x_{i}\;\right)-model\_estimated\_output(x_{i})$$

(3) $$fitness(x)=\frac{\sum_{x=0}^{n}err(x)}{n}$$

(4) $$leader=r_{1}\times x\left(leader_{pos},j\right)$$

where $r_{1}$ denotes a random number between [0, 1], x is the previous position of the leader, $leader_{pos}$ denotes the old position of the leader, and *j* refers to “each diminution”. After the leader’s position is updated, all members (or search agents) update their positions using Equation (5).

(5) $$x\left(i,j\right)=\left(x\left(i,j\right)-\left(circ\times x\left(i,j\right)+leader\right)\right)$$

where *circ* denotes circular motion, it is calculated as follows to try to replicate the circle system in Equation (6):

(6) $$circ=sqrt(n_{1}^{2}+n_{2}^{2})$$

(7) $$n_{1}=r_{2}\times \cos\left(ang\right)$$

(8) $$n_{2}=r_{2}\times \sin\left(ang\right)$$

where $r_{2}$ is the radius and is a random number between [0, 1], and ang denotes the angle of a move and is a random value between [0, 360] in Equation (7) and (8). Every generation, the ang is updated as well, and this update is based on the lower and upper bounds of the variables, where *lb* and *ub* are the lower and upper bands of the random number generator, respectively.

(9) $$dalta=random\left[lb,ub\right]$$

(10) ang=ang+dalta

If the value of the output grows larger than 360, or less than 0, the angle (*ang*) can be set to 360 or 0 to keep it within the desired range.

Algorithm 1 explains the RHSOFS pseudo-code. The RHSOFS begins by producing a binary population of P agents at random and examining all of the features. For each instance of the population to be studied, create a feature subset equal to 1. These chosen attributes are fed into classification models in order to calculate fitness value. Equation (2) calculates the *err(x)* by the differences between an actual and a predicted value of the model, where *x* = 1, 2, …, *n* and *n* is the number of testing observations.

The model’s fitness is calculated by dividing the sum of errors by the number of observations, as shown in Equation (3). The algorithm then attempts to update the position of the Leader according to Equation (4) and the position of each search agent according to Equation (5). Then, using Equation (3), determine each search agent’s new fitness. According to Equations (9) and (10), this algorithm progresses toward angle updating. The bestX persons are those who have the lowest fitness value. This algorithm then tries to update each search agent’s position in accordance with Equation (5). 

The new individuals are chosen only if their new fitness value is greater than or equal to their prior fitness value, and their new fitness value is flipped. For the next generation, only those with the lowest fitness value are chosen. Finally, the algorithm selects the most suitable candidates.
**Algorithm 1** Proposed RHSOFS AlgorithmCreate an initial population of 0 & 1 of P agents randomly.Set the dimension of the problem, *D* = P, where P is the number of agents.Set Low to 1 and High to *D*, where Low and High refer to the low and high dimensions, respectively.Generate the value of *r1* and *r2***,** where *r1* is a random number (0, 1) and *r2* is a random radius (0, 360).Generate training and testing data.Set max_iter = maximum number of iterations.Calculate each agent’s fitness using Equation (3).Set Leader = the best agent.Set t = 1.while (t < max_iter) for (i = 1 to n) do  Update Leader position, according to Equation (4).  Update the position of each search agent according to Equation (5).  Calculate Newfitness of each search agent using Equation (3).  Select the best member of the population → bestX = X (min (fitness))  Update the angle according to Equations (9) and (10).  If (Newfitness (i) <= fitness (i); then   Update the position of each search agent according to Equation (5).   fitness (i) = Newfitness (i).  end if end for t = t + 1.end while  Return the best agent

## 5. Experimental and Statistical Result Analysis and Discussion

To evaluate and examine the performance and effectiveness of the proposed FS approach, called RHSOFS, we have compared it with other useful approaches such as DEFS, GAFS, PSOFS, and ACOFS. Numerical experiments have been conducted on a real-world credit card fraud dataset using a range of data mining approaches to test the efficiency of the presented approach. The stratified cross-validation approaches have been used to create ten identical datasets due to a shortage of real credit card fraud datasets. The stratified cross-validation (SCV) method, which is related to the k-fold cross-validation method, is used to provide training as well as test indices for dividing the entire dataset into train and test sets by keeping the percentage of samples for each class the same for each fold. The stratified cross-validation approach is used for classification problems and when the dataset is imbalanced. Imbalanced datasets can create overfit results. The SCV technique creates new datasets by preserving the target class ratio in each fold the same as it is in the full dataset rather than randomly splitting the entire dataset.

Steps followed to create ten identical datasets using a stratified cross-validation approach:

1. Initially, the entire original dataset has been randomly shuffled;
2. The randomly shuffled dataset is split into k folds (we have set k = 10);
3. For each fold, the training dataset samples are selected by using stratified sampling in order to maintain the class distribution as per the original datasets. The test set is formed from the remaining datasets. This process is repeated for each fold.

In September 2013, European cardholders using credit cards collected the dataset in two days, and it is now available via the ULB machine learning group. This dataset is transformed using the PCA approach, which has 28 principal components or features spanning from V1 to V28. However, only 30 are included in the evaluation. There are a total of 284,807 transactions in this dataset, with 492 of them being fraud transactions, making it highly lopsided and skewed toward fraud [32]. 

The tests are performed on a PC with the following specifications: a 1.60 GHz Intel Core i5-8250U processor and 8 GB of RAM. Matlab 2014b is used to implement these approaches. The size of the population (Pop) and the maximum number of possible generations (MaxGen) are the variables that are used to train and test the model in the experiment.

The values of Pop and MaxGen have been set to 10 and 100, respectively, for the optimum overall performance. This section examines the performance of DEFS, GAFS, PSOFS, ACOFS, and RHSOFS techniques with respect to the number of selected features and the accuracy achieved by each technique. Because the procedures are stochastic, ten trials were carried out with a random sample population. The average classification accuracy for each dataset and FS approach is shown in Table 1. The results suggest that the proposed RHSOFS approach can achieve greater values than other approaches, such as DEFS, GAFS, PSOFS, and ACOFS, with the best optimal features. There are significantly fewer selected features compared to the original input features, as shown in Table 2. In comparison to other existing approaches (DEFS, GAFS, PSOFS, and ACOFS), which produce similar accuracy values with few variations, the new RHSOFS methodology produces significant accuracy results for almost all datasets using Equation (11).

(11) $$\mathrm{Accuracy}=\frac{TP+TN}{TP+TN+FP+FN}$$

**Figure 4:**
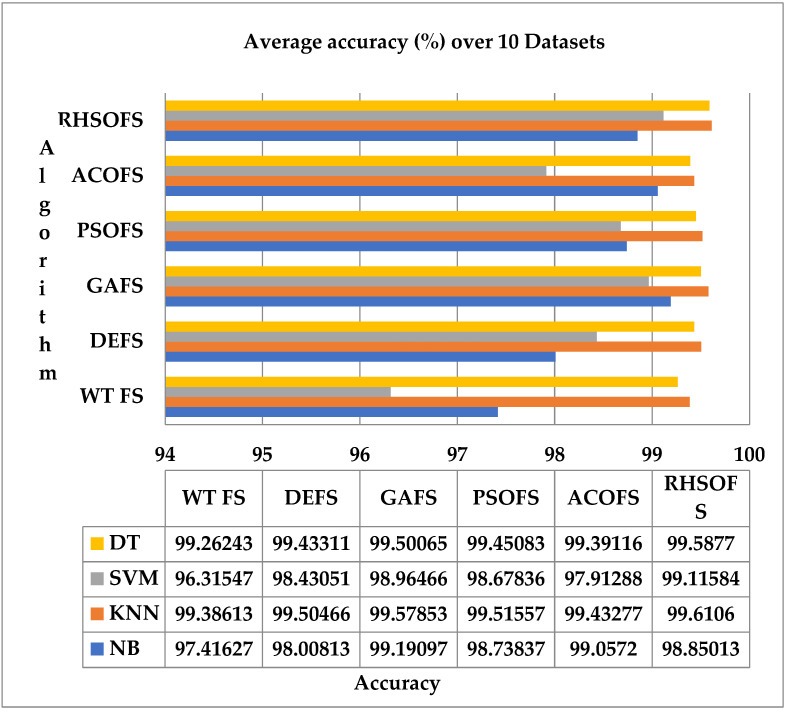
Average accuracy (%) of all models over 10 datasets.

**Figure 5:**
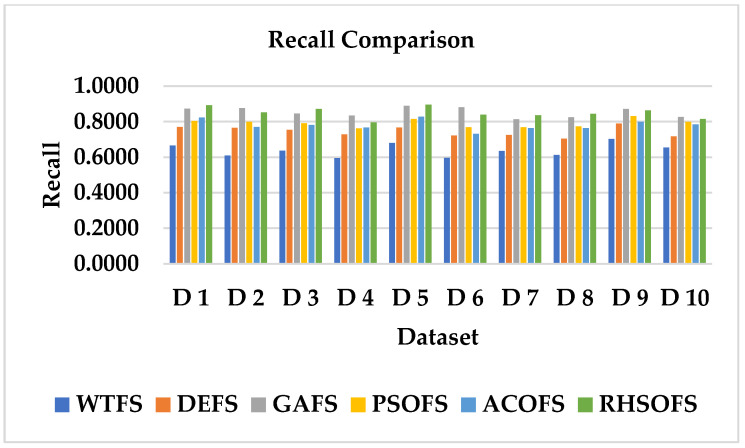
Recall comparison of all models over 10 datasets.

The average accuracy (%) over the 10 datasets using NB, KNN, SVM, and DT classifiers has been studied and analyzed, as depicted in Figure 4, applied over the FS approaches such as DEFA, GAFS, PSOFS, ACOFS, and RHSOFS, respectively. The recall comparison, as shown in Figure 5, identifies the effectiveness of the proposed method by comparing the recall values of all the models over each dataset. The results show that the proposed RHSOFS approaches have outperformed other FS approaches.

The goal of FS approaches such as DEFA, GAFS, PSOFS, ACOFS, and RHSOFS is to locate the best features and cut down on execution time to build a reliable credit card fraud detection system. For most FS algorithms, controlling elements such as the size of the population and the number of iterations are considered 10 and 100, respectively. Only approach-specific regulating parameters, such as probabilities of mutation, crossover, selection operators, initial weighting factors, and cognitive and social scaling factors, differ across all other FS techniques.

Table 3 and Table 4 show performance indicators for all datasets, including precision, recall, f1-score, Matthews correlation coefficient (MCC), and specificity with and without the feature (WTFS) subset selection. Finally, the proposed approach has a significantly lower number of selected features than previous approaches, resulting in a significant improvement in classification accuracy. Different performance measures from the confusion matrix include classification accuracy (Equation (11)), precision (Equation (12)), recall (Equation (13)), f-measure (Equation (14)), MCC (Equation (15)), and specificity (Equation (16)). True positive, true negative, false positive, and false negative are represented by the letters *TP*, *TN*, *FP*, and *FN*, respectively.

(12) $$Precision=\frac{TP}{TP+FP}$$

(13) $$Recall=\frac{TP}{TP+TN}$$

(14) $$F-measure=2\times Precision\times \frac{Recall}{Precision+Recall}$$

(15) $$MCC=\frac{TP\times TN-FP\times FN}{\sqrt{\left(TP+FP\right)\left(TP+FN\right)\left(TN+FP\right)\left(TN+FN\right)}}$$

(16) $$Specificity=\frac{TN}{TN+FP}$$

The performance of the proposed RHSOFS model has been compared to that of other models using statistical analysis [14]. The Friedman test is a non-parametric statistical method for analyzing the results of models. Friedman proposes two hypotheses (H0 and H1), with H0 implying that there is no significant variance among all approaches and that all approaches are considered equivalent, whereas H1 implies the opposite. The Friedman test is one of the best techniques to determine the importance of statistics across all approaches, and an individual approach is rated based on its accuracy.

This test has been conducted by considering the smallest number is assigned the highest rank, while the highest number is assigned the lowest rank. Table 5 shows the average rank of many models in relation to four different classifiers (NB, KNN, DT, and SVM) using Equation (17). It divides the number of classifiers by the total of all of their ranks. Similarly, Equation (18) is used to determine the average rank of various models in relation to the datasets shown in Table 6. The average rankings of all models are calculated by dividing the total number of datasets by the summation of the average rank (AR) of all models. Finally, the *AR* of all models represents RHSOFS (*AR1* = *1.525*), GAFS (*AR2* = *1.725*), PSOFS (*AR3* = *3.2*), ACOFS (*AR4* = *3.85*), DEFS (*AR5* = *3.975*), and WTFS (*AR6* = *5.825*).

(17) $$AR_{Models}=\frac{Summation\;of\;rank\;of\;all\;classifiers}{No.of\;classifiers}$$

(18) $$AR_{Datasets}=\frac{Summation\;of\;AR_{Models}}{No.of\;datasets}$$

(19) $$X_{F}^{2}=\left[\frac{12\times N}{P\left(P+1\right)}\right]\times \left[\sum_{j}AR^{2}-\frac{P\times {\left(P+1\right)}^{2}}{4}\right]$$

(20) $$F_{F}=\frac{\left(N-1\right)\times X_{F}^{2}}{N\times \left(P-1\right)-X_{F}^{2}}$$

Using Equation (19), the Friedman test statistic has a chi-square ($X_{F}^{2}$) distribution with (*P* − 1) degrees of freedom. Whereas Equation (20) yields a value of 5.442917 for the Friedman test statistic (*F_F_*), where *N* is the number of datasets and *P* denotes the number of models that were employed in this experiment, The number of datasets used in this study is ten, the number of models employed is six, and the threshold of significance of a is 0.05 with degrees of freedom (5, 45). The null hypothesis is rejected because the crucial value of *F_F_* is 2.42, which is smaller than the Friedman statistics of *F_F_* = 5.442917.

(21) $$z=\frac{\left(AR_{i}-AR_{j}\right)}{\sqrt{P(P+1)/6N}}$$

The number of algorithms employed in this experiment is *P*, *z* is used to calculate the *z*-score value using Equation (21), and the number of datasets is *N*. *AR_i_* and *AR_j_* denote the average rank of the *i*th and *j*th models, respectively. All the models are compared to the suggested model using the *z*-value, *p*-value, and *α*/*(P − i),* and the results are shown in Table 7. 

There may be some insignificant, duplicated, or noisy data in the feature set, which increases processing time while also affecting the model’s performance. Only optimal features are processed by the model, and all unwanted, redundant, and noisy characteristics are eliminated. These relevant features boost the model’s performance while cutting down on computation time. FS methods, on the other hand, pick a subset of significant and relevant original features. This paper compares the model’s performance, including NB, KNN, SVM, and DT, using wrapper-based FS methods such as DEFS, GAFS, PSOFS, ACOFS, and RHSOFS. To compute to select the best features, modeling FS processes can be used in two ways: first, to choose fixed optimum features, and second, to select variable optimum features. It is a difficult task to determine the set number of optimum features for all models in the fixed optimal FS technique. The dataset used in the experiment was already processed using the PCA method, which contains 28 principal components or features ranging from V1 to V28. There may be some features that are duplicated. Another difficult task is to reduce the redundant features and replace redundant features with the next most important features. On the other hand, distinct FS techniques based on several optimization algorithms select the fewest number of variable optimum features. Individual features are assigned a value of 1 or 0 in this method. The goal of the presented RHSOFS approach is to remove irrelevant features by selecting the most relevant and appropriate features to improve the model’s performance. The number of relevant features used is determined by the performance of the optimization methods. Furthermore, applying different optimization techniques does not guarantee that you will select the same number of optimum features.

Finally, the model has learned to use these selected relevant features gained by optimization procedures. The goal of this paper is to boost the model’s performance by optimizing the number of relevant features. A scale limit can often be provided based on the rank of the relevant features or the number of features to be chosen in a fixed optimal FS technique, ensuring that the predefined number of features is selected. The comparison is based on the best FS using different optimization algorithms.

## 6. Conclusions

To determine an optimal subset of features, a new unique FS technique depending on the RHO algorithm named RHSOFS, has been presented. The RHSOFS approach is used to explore the most relevant features by updating the irrelevant, redundant, and noisy features. Four classifiers, such as NB, KNN, SVM, and DT, have been employed on ten datasets for evaluating the efficacy of the proposed RHSOFS approach.

The proposed RHSOFS strategy effectively reduces duplicate features and outperforms existing approaches, according to the experimental data. In terms of classification accuracy and the number of characteristics chosen, all the models are compared. The data points that are insignificant, duplicated, or noisy should be removed. This could result in data loss, which is a drawback of this method. Certainly, the use of AI techniques for the prediction of future behaviors can have good results, although, in the field of cybercrime, it is more complex since it is trained with some acquired knowledge, not counting on the evolution in the learning of the offender. Although this article presents a very interesting proposal, we would recommend taking into account works such as “Evolution Oriented Monitoring oriented to Security Properties for Cloud Applications” in the sense of providing applications with the ability to evolve securely by integrating acquired knowledge. On the other hand, it would be interesting to study how to endow Trusted Computing-type trusted hardware with this type of intelligence so that they can provide hybrid hardware–software certification mechanisms in different scenarios, as proposed in “Software and Hardware Certification Techniques in a Combined Certification Model”. This proposed strategy could even be applied to a wide range of complex and different applications with many features, such as mail fraud detection, intrusion detection, and fake insurance analysis.

## Figures and Tables

**Table 1:** Classification accuracy (%) of all feature selection approaches.

Sl. No	Datasets	Feature Selection Algorithms	NB	KNN	DT	SVM	No. of Selected Features
1	D1	WT FS	97.02245	99.45030	99.40449	96.19789	28
		DEFS	97.97984	99.50069	99.47778	98.56619	21
		GAFS	99.03802	99.63353	99.45030	99.08383	14
		PSOFS	98.62574	99.60605	99.45030	98.80898	15
		ACOFS	98.80898	99.49611	99.49611	97.80119	14
		RHSOFS	98.85479	99.67934	99.63353	99.26706	13
2	D2	WTFS	97.70957	99.35868	99.03802	96.56436	28
		DEFS	98.57077	99.48847	99.48956	98.61658	17
		GAFS	99.40449	99.58772	99.54191	99.17545	13
		PSOFS	98.90060	99.51901	99.52665	98.80898	14
		ACOFS	99.30371	99.38158	99.31745	98.16766	14
		RHSOFS	98.99221	99.63353	99.58772	99.22126	12
3	D3	WT FS	97.75538	99.35868	99.45030	96.19789	28
		DEFS	98.45625	99.49611	99.53275	98.46541	18
		GAFS	99.31287	99.54191	99.58772	98.90060	12
		PSOFS	98.90060	99.51901	99.49611	98.68530	13
		ACOFS	99.17545	99.42281	99.54191	98.07604	13
		RHSOFS	98.94640	99.63353	99.63353	99.26706	11
4	D4	WT FS	97.11406	99.22126	99.22126	96.51855	28
		DEFS	98.10811	99.47320	99.39991	98.32341	17
		GAFS	99.31287	99.49611	99.40449	98.80898	12
		PSOFS	98.85479	99.37700	99.32203	98.50206	13
		ACOFS	98.80898	99.29913	99.26706	98.07604	12
		RHSOFS	98.85479	99.49611	99.45030	98.76317	12
5	D5	WT FS	97.48053	99.49611	99.12964	96.19789	28
		DEFS	97.83784	99.50611	99.31287	98.38754	24
		GAFS	99.08383	99.54191	99.31287	99.08383	12
		PSOFS	98.76317	99.52611	99.24416	98.67155	14
		ACOFS	98.97847	99.51611	99.26706	98.39670	12
		RHSOFS	98.80898	99.58772	99.45030	99.26706	12
6	D6	WT FS	97.11406	99.45030	99.35868	95.09849	28
		DEFS	97.59505	99.58772	99.57398	98.46541	21
		GAFS	99.45030	99.67934	99.67934	99.17545	14
		PSOFS	98.44251	99.61521	99.56809	98.76317	14
		ACOFS	99.08841	99.51647	99.40449	97.20568	15
		RHSOFS	98.71736	99.63353	99.67934	99.26706	13
7	D7	WT FS	97.80119	99.45030	99.35868	96.35822	28
		DEFS	98.27302	99.58772	99.39991	98.59826	20
		GAFS	99.08383	99.63353	99.63353	98.94640	14
		PSOFS	98.90060	99.59689	99.60081	98.48832	14
		ACOFS	99.30371	99.47778	99.45030	97.98443	12
		RHSOFS	98.90060	99.72515	99.67934	99.26706	12
8	D8	WT FS	97.15987	99.35868	99.40449	96.56894	28
		DEFS	97.73706	99.49611	99.49611	98.39212	21
		GAFS	99.22126	99.58772	99.58772	98.94640	13
		PSOFS	98.62574	99.50985	99.55718	98.75859	13
		ACOFS	99.13880	99.40449	99.54191	98.07604	14
		RHSOFS	99.17545	99.63353	99.67934	99.03802	12
9	D9	WT FS	97.89281	99.45030	99.35868	96.79340	28
		DEFS	98.13559	99.55209	99.56482	98.53413	21
		GAFS	99.03802	99.67934	99.54191	98.99221	13
		PSOFS	98.80898	99.60147	99.59536	98.62574	13
		ACOFS	99.23958	99.50069	99.49611	97.17820	12
		RHSOFS	98.90060	99.67934	99.67934	98.99221	12
10	D10	WT FS	97.11274	99.26673	98.90009	96.65903	28
		DEFS	97.38772	99.35839	99.08341	97.95600	22
		GAFS	98.96425	99.40422	99.26673	98.53346	13
		PSOFS	98.56095	99.28506	99.14757	98.67094	14
		ACOFS	98.72594	99.31256	99.12924	98.16682	11
		RHSOFS	98.35014	99.40422	99.40422	98.80843	10

**Table 2:** List of the subset of selected features for each dataset.

Datasets	Number of Selected Features	Subset of Selected Features
D1	13	3, 4, 6, 9, 11, 12, 14, 15, 16, 17, 18, 22, 25
D2	12	4, 6, 7, 9, 10, 11, 12, 13, 16, 17, 18, 22
D3	11	4, 5, 11, 12, 13, 15, 16, 17, 19, 25, 26
D4	12	3, 6, 11, 12, 13, 14, 15, 16, 17, 19, 24, 26
D5	12	3, 4, 5, 6, 9, 11, 12, 14, 15, 16, 18, 22
D6	13	4, 7, 9, 11, 12, 13, 14, 16, 17, 23, 24, 25, 25
D7	12	6, 11, 12, 13, 14, 15, 16, 18, 19, 22, 24, 26
D8	12	3, 4, 9, 10, 11, 14, 16, 18, 19, 24, 25, 26
D9	12	3, 4, 6, 9, 11, 12, 14, 16, 17, 18, 24, 26
D10	10	1, 6, 9, 13, 14, 15, 16, 18, 24, 28

**Table 3:** Recall, Precision, F1-score MCC, and Specificity for all approaches with NB and KNN classifier.

Sl. No	Dataset	FS Algorithm	NB					KNN				
			Recall	Precision	F1 Score	MCC	Specificity	Recall	Precision	F1 Score	MCC	Specificity
1	D 1	WTFS	0.4400	0.8302	0.5752	0.5918	0.9957	0.9020	0.8679	0.8846	0.8820	0.9967
		DEFS	0.5549	0.8491	0.6711	0.6772	0.9962	0.9287	0.8604	0.8932	0.8914	0.9965
		GAFS	0.7500	0.9057	0.8205	0.8194	0.9976	1.0000	0.8491	0.9184	0.9197	0.9963
		PSOFS	0.6575	0.9057	0.7619	0.7653	0.9976	0.9644	0.8698	0.9147	0.9139	0.9968
		ACOFS	0.9091	0.5660	0.6977	0.7122	0.9893	0.9375	0.8491	0.8911	0.8897	0.9963
		RHSOFS	0.7121	0.8868	0.7899	0.7891	0.9972	1.0000	0.8679	0.9293	0.9301	0.9967
2	D 2	WTFS	0.4684	0.8222	0.5968	0.6106	0.9962	0.8605	0.8222	0.8409	0.8379	0.9963
		DEFS	0.6131	0.8311	0.7057	0.7070	0.9964	0.9177	0.8259	0.8694	0.8680	0.9963
		GAFS	0.8636	0.8444	0.8539	0.8509	0.9967	0.9500	0.8444	0.8941	0.8936	0.9967
		PSOFS	0.6909	0.8444	0.7600	0.7584	0.9967	0.9177	0.8422	0.8783	0.8767	0.9967
		ACOFS	0.8341	0.8267	0.8304	0.8268	0.9964	0.8741	0.8178	0.8450	0.8423	0.9962
		RHSOFS	0.7170	0.8444	0.7755	0.7731	0.9967	0.9512	0.8667	0.9070	0.9061	0.9972
3	D 3	WTFS	0.4605	0.8140	0.5882	0.6025	0.9962	0.8372	0.8372	0.8372	0.8339	0.9967
		DEFS	0.5746	0.8326	0.6800	0.6845	0.9966	0.9082	0.8279	0.8662	0.8646	0.9965
		GAFS	0.8333	0.8140	0.8235	0.8201	0.9963	0.9231	0.8372	0.8780	0.8768	0.9967
		PSOFS	0.6863	0.8140	0.7447	0.7419	0.9962	0.9177	0.8302	0.8718	0.8705	0.9966
		ACOFS	0.7990	0.7767	0.7877	0.7836	0.9955	0.8707	0.8302	0.8500	0.8473	0.9966
		RHSOFS	0.7000	0.8140	0.7527	0.7496	0.9962	0.9487	0.8605	0.9024	0.9017	0.9972
4	D 4	WTFS	0.3956	0.8182	0.5333	0.5572	0.9962	0.8140	0.7955	0.8046	0.8007	0.9958
		DEFS	0.5199	0.8000	0.6303	0.6362	0.9958	0.9392	0.7898	0.8580	0.8587	0.9957
		GAFS	0.8222	0.8409	0.8315	0.8280	0.9967	0.9459	0.7955	0.8642	0.8650	0.9958
		PSOFS	0.7021	0.7500	0.7253	0.7198	0.9949	0.8918	0.7864	0.8357	0.8343	0.9956
		ACOFS	0.8750	0.4773	0.6176	0.6413	0.9893	0.8458	0.7977	0.8211	0.8178	0.9958
		RHSOFS	0.6792	0.8182	0.7423	0.7398	0.9962	0.9231	0.8182	0.8675	0.8665	0.9963
5	D 5	WTFS	0.5000	0.8000	0.6154	0.6209	0.9947	0.9583	0.8364	0.8932	0.8928	0.9958
		DEFS	0.5516	0.7582	0.6386	0.6362	0.9937	0.9512	0.8357	0.8897	0.8890	0.9957
		GAFS	0.8431	0.7818	0.8113	0.8072	0.9944	0.9707	0.8436	0.9027	0.9027	0.9960
		PSOFS	0.7333	0.8000	0.7652	0.7596	0.9948	0.9314	0.8482	0.8879	0.8861	0.9960
		ACOFS	0.8399	0.7345	0.7837	0.7803	0.9932	0.9400	0.8545	0.8952	0.8937	0.9962
		RHSOFS	0.7458	0.8000	0.7719	0.7663	0.9948	0.9792	0.8545	0.9126	0.9127	0.9963
6	D 6	WTFS	0.4200	0.8936	0.5714	0.6015	0.9976	0.8571	0.8936	0.8750	0.8724	0.9977
		DEFS	0.4692	0.8915	0.6148	0.6370	0.9976	0.9130	0.8936	0.9032	0.9012	0.9977
		GAFS	0.8571	0.8936	0.8750	0.8724	0.9977	0.9545	0.8936	0.9231	0.9220	0.9977
		PSOFS	0.5915	0.8936	0.7119	0.7201	0.9976	0.9196	0.9000	0.9097	0.9078	0.9978
		ACOFS	0.7561	0.8511	0.8008	0.7976	0.9967	0.8814	0.8960	0.8886	0.8862	0.9977
		RHSOFS	0.6557	0.8511	0.7407	0.7408	0.9967	0.9333	0.8936	0.9130	0.9114	0.9977
7	D 7	WTFS	0.4886	0.9348	0.6418	0.6672	0.9986	0.8696	0.8696	0.8696	0.8668	0.9972
		DEFS	0.5553	0.9065	0.6887	0.7020	0.9980	0.9044	0.8995	0.9019	0.8998	0.9978
		GAFS	0.7241	0.9130	0.8077	0.8087	0.9981	0.9318	0.8913	0.9111	0.9095	0.9977
		PSOFS	0.6774	0.9130	0.7778	0.7813	0.9981	0.9026	0.9065	0.9046	0.9025	0.9980
		ACOFS	0.8348	0.8348	0.8348	0.8312	0.9964	0.8681	0.8870	0.8774	0.8748	0.9976
		RHSOFS	0.6897	0.8696	0.7692	0.7691	0.9972	0.9348	0.9348	0.9348	0.9334	0.9986
8	D 8	WTFS	0.4022	0.8409	0.5441	0.5702	0.9967	0.8409	0.8409	0.8409	0.8376	0.9967
		DEFS	0.4662	0.8455	0.6010	0.6182	0.9968	0.8966	0.8477	0.8715	0.8693	0.9969
		GAFS	0.7755	0.8636	0.8172	0.8145	0.9972	0.9070	0.8864	0.8966	0.8945	0.9977
		PSOFS	0.6207	0.8182	0.7059	0.7060	0.9962	0.9091	0.8409	0.8737	0.8719	0.9967
		ACOFS	0.7763	0.8045	0.7902	0.7859	0.9960	0.8605	0.8409	0.8506	0.8476	0.9967
		RHSOFS	0.7600	0.8636	0.8085	0.8060	0.9972	0.9500	0.8636	0.9048	0.9040	0.9972
9	D 9	WTFS	0.5714	0.8814	0.6933	0.7002	0.9967	0.8983	0.8983	0.8983	0.8955	0.9972
		DEFS	0.6060	0.8864	0.7199	0.7244	0.9968	0.9284	0.9040	0.9160	0.9138	0.9973
		GAFS	0.7714	0.9153	0.8372	0.8355	0.9976	1.0000	0.8814	0.9369	0.9373	0.9967
		PSOFS	0.7260	0.8983	0.8030	0.8018	0.9972	0.9564	0.8932	0.9238	0.9223	0.9970
		ACOFS	0.8545	0.8661	0.8603	0.8564	0.9963	0.9227	0.8898	0.9060	0.9035	0.9969
		RHSOFS	0.7465	0.8983	0.8154	0.8135	0.9972	0.9643	0.9153	0.9391	0.9378	0.9976
10	D 10	WTFS	0.4632	0.7857	0.5828	0.5903	0.9943	0.9167	0.7857	0.8462	0.8450	0.9944
		DEFS	0.4944	0.7875	0.6074	0.6120	0.9943	0.9375	0.8036	0.8654	0.8648	0.9948
		GAFS	0.7961	0.8018	0.7989	0.7936	0.9948	0.9388	0.8214	0.8762	0.8752	0.9953
		PSOFS	0.6887	0.8018	0.7409	0.7358	0.9948	0.9122	0.7982	0.8514	0.8498	0.9947
		ACOFS	0.7722	0.7143	0.7421	0.7362	0.9925	0.9020	0.8214	0.8598	0.8573	0.9953
		RHSOFS	0.6429	0.8036	0.7143	0.7106	0.9948	0.9388	0.8214	0.8762	0.8752	0.9953

**Table 4:** Recall, Precision, F1-score MCC, and Specificity for all approaches with DT and SVM classifier.

Sl. No	Dataset	FS Algorithm	DT					SVM				
			Recall	Precision	F1 Score	MCC	Specificity	Recall	Precision	F1 Score	MCC	Specificity
1	D 1	WTFS	0.9348	0.8113	0.8687	0.8679	0.9953	0.3852	0.8545	0.5311	0.5590	0.9961
		DEFS	0.9407	0.8377	0.8862	0.8851	0.9960	0.6575	0.8547	0.7432	0.7427	0.9964
		GAFS	0.9362	0.8302	0.8800	0.8789	0.9958	0.8070	0.8364	0.8214	0.8169	0.9958
		PSOFS	0.8837	0.8444	0.8636	0.8611	0.9967	0.7077	0.8679	0.7797	0.7779	0.9967
		ACOFS	0.9200	0.8679	0.8932	0.8910	0.9967	0.5275	0.9057	0.6667	0.6819	0.9976
		RHSOFS	0.9787	0.8679	0.9200	0.9199	0.9967	0.8776	0.8113	0.8431	0.8401	0.9953
2	D 2	WTFS	0.7400	0.8222	0.7789	0.7752	0.9962	0.3684	0.9333	0.5283	0.5746	0.9986
		DEFS	0.9100	0.8349	0.8709	0.8691	0.9965	0.6205	0.8467	0.7162	0.7183	0.9967
		GAFS	0.9268	0.8444	0.8837	0.8824	0.9967	0.7647	0.8667	0.8125	0.8100	0.9972
		PSOFS	0.9194	0.8444	0.8803	0.8787	0.9967	0.6667	0.8444	0.7451	0.7445	0.9967
		ACOFS	0.8397	0.8267	0.8331	0.8297	0.9964	0.5333	0.8889	0.6667	0.6806	0.9976
		RHSOFS	0.9500	0.8444	0.8941	0.8936	0.9967	0.7917	0.8444	0.8172	0.8137	0.9967
3	D 3	WTFS	0.9189	0.7907	0.8500	0.8497	0.9958	0.3276	0.8837	0.4780	0.5249	0.9976
		DEFS	0.9432	0.8116	0.8725	0.8727	0.9962	0.5874	0.8023	0.6782	0.6791	0.9959
		GAFS	0.9474	0.8372	0.8889	0.8886	0.9967	0.6792	0.8372	0.7500	0.7487	0.9967
		PSOFS	0.9286	0.8062	0.8631	0.8627	0.9961	0.6284	0.8140	0.7092	0.7088	0.9962
		ACOFS	0.9459	0.8140	0.8750	0.8752	0.9963	0.5068	0.8605	0.6379	0.6521	0.9972
		RHSOFS	0.9730	0.8372	0.9000	0.9008	0.9967	0.8649	0.7442	0.8000	0.7986	0.9949
4	D 4	WTFS	0.8140	0.7955	0.8046	0.8007	0.9958	0.3519	0.8636	0.5000	0.5385	0.9971
		DEFS	0.8951	0.7955	0.8424	0.8408	0.9958	0.5591	0.7955	0.6567	0.6589	0.9958
		GAFS	0.8974	0.7955	0.8434	0.8419	0.9958	0.6731	0.7955	0.7292	0.7258	0.9958
		PSOFS	0.8578	0.7955	0.8255	0.8226	0.9958	0.5956	0.8000	0.6828	0.6830	0.9959
		ACOFS	0.8333	0.7955	0.8140	0.8104	0.9958	0.5143	0.8182	0.6316	0.6400	0.9962
		RHSOFS	0.9211	0.7955	0.8537	0.8532	0.9958	0.6604	0.7955	0.7216	0.7186	0.9958
5	D 5	WTFS	0.8750	0.7636	0.8155	0.8131	0.9939	0.3852	0.8545	0.5311	0.5590	0.9961
		DEFS	0.9217	0.7948	0.8536	0.8525	0.9947	0.6402	0.8218	0.7197	0.7175	0.9954
		GAFS	0.9348	0.7818	0.8515	0.8515	0.9944	0.8070	0.8364	0.8214	0.8169	0.9958
		PSOFS	0.8889	0.8000	0.8421	0.8395	0.9948	0.7083	0.8036	0.7530	0.7478	0.9949
		ACOFS	0.8980	0.8000	0.8462	0.8439	0.9948	0.6316	0.8727	0.7328	0.7349	0.9967
		RHSOFS	0.9574	0.8182	0.8824	0.8824	0.9953	0.8980	0.8000	0.8462	0.8439	0.9948
6	D 6	WTFS	0.8113	0.9149	0.8600	0.8583	0.9981	0.2973	0.9362	0.4513	0.5124	0.9985
		DEFS	0.9089	0.8915	0.9001	0.8980	0.9976	0.5939	0.9085	0.7183	0.7277	0.9980
		GAFS	0.9545	0.8936	0.9231	0.9220	0.9977	0.7544	0.9149	0.8269	0.8268	0.9981
		PSOFS	0.9122	0.8845	0.8981	0.8961	0.9975	0.6515	0.9149	0.7611	0.7664	0.9981
		ACOFS	0.8542	0.8723	0.8632	0.8602	0.9972	0.4314	0.9362	0.5906	0.6252	0.9986
		RHSOFS	0.9762	0.8723	0.9213	0.9212	0.9972	0.7925	0.8936	0.8400	0.8378	0.9977
7	D 7	WTFS	0.8200	0.8913	0.8542	0.8517	0.9977	0.3608	0.9435	0.5219	0.5712	0.9987
		DEFS	0.8297	0.9000	0.8634	0.8611	0.9978	0.6122	0.9130	0.7330	0.7414	0.9981
		GAFS	0.9130	0.9130	0.9130	0.9112	0.9981	0.6825	0.9348	0.7890	0.7939	0.9986
		PSOFS	0.8967	0.9161	0.9063	0.9043	0.9982	0.5942	0.8913	0.7130	0.7209	0.9976
		ACOFS	0.8400	0.9130	0.8750	0.8730	0.9981	0.5119	0.9348	0.6615	0.6837	0.9986
		RHSOFS	0.9333	0.9130	0.9231	0.9215	0.9981	0.7885	0.8913	0.8367	0.8346	0.9977
8	D 8	WTFS	0.8444	0.8636	0.8539	0.8509	0.9972	0.3602	0.9045	0.5152	0.5587	0.9980
		DEFS	0.8837	0.8636	0.8736	0.8711	0.9972	0.5669	0.8568	0.6824	0.6896	0.9970
		GAFS	0.9268	0.8636	0.8941	0.8926	0.9972	0.6909	0.8636	0.7677	0.7673	0.9972
		PSOFS	0.9187	0.8561	0.8863	0.8846	0.9970	0.6435	0.8614	0.7366	0.7386	0.9971
		ACOFS	0.9048	0.8636	0.8837	0.8816	0.9972	0.5135	0.8636	0.6441	0.6576	0.9972
		RHSOFS	0.9512	0.8864	0.9176	0.9166	0.9977	0.7170	0.8636	0.7835	0.7822	0.9972
9	D 9	WTFS	0.8814	0.8814	0.8814	0.8781	0.9967	0.4560	0.9661	0.6196	0.6519	0.9990
		DEFS	0.9508	0.8847	0.9166	0.9150	0.9968	0.6692	0.9051	0.7695	0.7713	0.9973
		GAFS	0.9455	0.8814	0.9123	0.9105	0.9967	0.7681	0.8983	0.8281	0.8256	0.9972
		PSOFS	0.9547	0.8927	0.9226	0.9211	0.9970	0.6883	0.8983	0.7794	0.7797	0.9972
		ACOFS	0.9286	0.8814	0.9043	0.9021	0.9967	0.4885	0.9390	0.6427	0.6662	0.9983
		RHSOFS	0.9643	0.9153	0.9391	0.9378	0.9976	0.7761	0.8814	0.8254	0.8220	0.9967
10	D 10	WTFS	0.8077	0.7500	0.7778	0.7727	0.9934	0.4282	0.9000	0.5803	0.6078	0.9973
		DEFS	0.8614	0.7661	0.8110	0.8077	0.9939	0.5714	0.8143	0.6716	0.6724	0.9951
		GAFS	0.9000	0.8036	0.8491	0.8467	0.9948	0.6667	0.8571	0.7500	0.7488	0.9962
		PSOFS	0.8816	0.7714	0.8229	0.8204	0.9940	0.7143	0.8036	0.7563	0.7509	0.9948
		ACOFS	0.8627	0.7857	0.8224	0.8189	0.9944	0.5976	0.8750	0.7101	0.7147	0.9967
		RHSOFS	0.9216	0.8393	0.8785	0.8765	0.9958	0.7586	0.7857	0.7719	0.7659	0.9944

**Table 5:** Rank of all FS models.

Sl. No	Dataset	FS Algorithm		Accuracy				Avg. Rank	
				NB	KNN	DT	SVM		
1	D 1	WTFS		97.02245 (6)	99.45030 (6)	99.40449 (5)	96.19789 (6)	5.75	
		DEFS		97.97984 (5)	99.50069 (4)	99.47778 (3)	98.56619 (4)	4	
		GAFS		99.03802 (1)	99.63353 (2)	99.45030 (4)	99.08383 (2)	2.25	
		PSOFS		98.62574 (4)	99.60605 (3)	99.45030 (4)	98.80898 (3)	3.5	
		ACOFS		98.80898 (3)	99.49611 (5)	99.49611 (2)	97.80119 (5)	3.75	
		RHSOFS		98.85479 (2)	99.67934 (1)	99.63353 (1)	99.26706 (1)	1.25	
2	D 2	WTFS		97.70957 (6)	99.35868 (6)	99.03802 (6)	96.56436 (6)	6	
		DEFS		98.57077 (5)	99.48847 (4)	99.48956 (4)	98.61658 (4)	4.25	
		GAFS		99.40449 (1)	99.58772 (2)	99.54191 (2)	99.17545 (2)	1.75	
		PSOFS		98.90060 (4)	99.51901 (3)	99.52665 (3)	98.80898 (3)	3.25	
		ACOFS		99.30371 (2)	99.38158 (5)	99.31745 (5)	98.16766 (5)	4.25	
		RHSOFS		98.99222 (3)	99.63353 (1)	99.58772 (1)	99.22126 (1)	1.5	
3	D 3	WTFS		97.75538 (6)	99.35868 (6)	99.45030 (6)	96.19789 (6)	6	
		DEFS		98.45625 (5)	99.49611 (4)	99.53275 (4)	98.46541 (4)	4.25	
		GAFS		99.31287 (1)	99.54191 (2)	99.58772 (2)	98.90060 (2)	1.75	
		PSOFS		98.90060 (4)	99.51901 (3)	99.49611 (5)	98.68530 (3)	3.75	
		ACOFS		99.17545 (2)	99.42281 (5)	99.54191 (3)	98.07604 (5)	3.75	
		RHSOFS		98.94640 (3)	99.63353 (1)	99.63353 (1)	99.26706 (1)	1.5	
4	D 4	WTFS		97.11406 (5)	99.22126 (5)	99.22126 (6)	96.51855 (6)	6	
		DEFS		98.10811 (4)	99.47320 (2)	99.39991 (3)	98.32341 (4)	3.25	
		GAFS		99.31287 (1)	99.49611 (1)	99.40449 (2)	98.80898 (1)	1.25	
		PSOFS		98.85479 (2)	99.37700 (3)	99.32203 (4)	98.50206 (3)	3	
		ACOFS		98.80898 (3)	99.29913 (4)	99.26706 (5)	98.07604 (5)	4.25	
		RHSOFS		98.85479 (2)	99.49611 (1)	99.45030 (1)	98.76317 (2)	1.5	
5	D 5	WTFS		97.48053 (6)	99.49611 (6)	99.12964 (5)	96.19789 (6)	5.75	
		DEFS		97.83784 (5)	99.50611 (5)	99.31287 (2)	98.38754 (5)	4.25	
		GAFS		99.08383 (1)	99.54191 (2)	99.31287 (2)	99.08383 (2)	1.75	
		PSOFS		98.76317 (4)	99.52611 (3)	99.24416 (4)	98.67155 (3)	3.5	
		ACOFS		98.97847 (2)	99.51611 (4)	99.26706 (3)	98.39670 (4)	3.25	
		RHSOFS		98.80898 (3)	99.58772 (1)	99.45030 (1)	99.26706 (1)	1.5	
6	D 6	WTFS		97.11406 (6)	99.45030 (6)	99.35868 (5)	95.09849 (6)	5.75	
		DEFS		97.59505 (5)	99.58772 (4)	99.57398 (2)	98.46541 (4)	3.75	
		GAFS		99.45030 (1)	99.67934 (1)	99.67934 (1)	99.17545 (2)	1.25	
		PSOFS		98.44251 (2)	99.61521 (3)	99.56809 (3)	98.76317 (3)	2.75	
		ACOFS		99.08841 (3)	99.51647 (5)	99.40449 (4)	97.20568 (5)	4.25	
		RHSOFS		98.71736 (4)	99.63353 (2)	99.67934 (1)	99.26706 (1)	2	
7	D 7	WTFS		97.80119 (5)	99.45030 (6)	99.35868 (6)	96.35822 (6)	5.75	
		DEFS		98.27302 (4)	99.58772 (3)	99.39991 (5)	98.59826 (3)	3.75	
		GAFS		99.08383 (2)	99.63353 (2)	99.63353 (2)	98.94640 (2)	2	
		PSOFS		98.90060 (3)	99.59689 (4)	99.60081 (3)	98.48832 (4)	3.5	
		ACOFS		99.30371 (1)	99.47778 (5)	99.45030 (4)	97.98443 (5)	3.75	
		RHSOFS		98.90060 (3)	99.72515 (1)	99.67934 (1)	99.26706 (1)	1.5	
8	D 8	WTFS		97.15987 (6)	99.35868 (6)	99.40449 (6)	96.56894 (6)	6	
		DEFS		97.73706 (5)	99.49611 (4)	99.49611 (5)	98.39212 (4)	4.5	
		GAFS		99.22126 (1)	99.58772 (2)	99.58772 (2)	98.94640 (2)	1.75	
		PSOFS		98.62574 (4)	99.50985 (3)	99.55718 (3)	98.75859 (3)	3.25	
		ACOFS		99.13880 (3)	99.40449 (5)	99.54191 (4)	98.07604 (5)	4.25	
		RHSOFS		99.17545 (2)	99.63353 (1)	99.67934 (1)	99.03802 (1)	1.25	
9	D 9	WTFS		97.89281 (6)	99.45030 (5)	99.35868 (6)	96.79340 (5)	5.5	
		DEFS		98.13559 (5)	99.55209 (3)	99.56482 (3)	98.53413 (3)	3.5	
		GAFS		99.03802 (1)	99.67934 (1)	99.54191 (4)	98.99221 (1)	1.75	
		PSOFS		98.80898 (4)	99.60147 (2)	99.59536 (2)	98.62574 (2)	2.5	
		ACOFS		99.23958 (2)	99.50069 (4)	99.49611 (5)	97.17820 (4)	3.75	
		RHSOFS		98.90060 (3)	99.67934 (1)	99.67934 (1)	98.99221 (1)	1.5	
10	D 10	WTFS		97.11274 (6)	99.26672 (5)	98.90009 (6)	96.65903 (6)	5.75	
		DEFS		97.38772 (5)	99.35839 (2)	99.08341 (5)	97.95600 (5)	4.25	
		GAFS		98.96425 (1)	99.40422 (1)	99.26672 (2)	98.53346 (3)	1.75	
		PSOFS		98.56095 (3)	99.28506 (4)	99.14757 (3)	98.67094 (2)	3	
		ACOFS		98.72594 (2)	99.31256 (3)	99.12924 (4)	98.16682 (4)	3.25	
		RHSOFS		98.35014 (4)	99.40422 (1)	99.40422 (1)	98.80843 (1)	1.75	

**Table 6:** Average rank of all models.

Dataset	WTFS	DEFS	GAFS	PSOFS	ACOFS	RHSOFS
D 1	5.75	4	2.25	3.5	3.75	1.25
D 2	6	4.25	1.75	3.25	4.25	1.5
D 3	6	4.25	1.75	3.75	3.75	1.5
D 4	6	3.25	1.25	3	4.25	1.5
D 5	5.75	4.25	1.75	3.5	3.25	1.5
D 6	5.75	3.75	1.25	2.75	4.25	2
D 7	5.75	3.75	2	3.5	3.75	1.5
D 8	6	4.5	1.75	3.25	4.25	1.25
D 9	5.5	3.5	1.75	2.5	3.75	1.5
D 10	5.75	4.25	1.75	3	3.25	1.75
Average	5.825 (6)	3.975 (5)	1.725 (2)	3.2 (3)	3.85 (4)	1.525 (1)

**Table 7:** Holm Test.

Sl. No	FS Algorithms	*z*-Values	*p*-Values	*α*/*(P − i)*
1	RHSOFS: WTFS	5.139	0.00001	0.01
2	RHSOFS: DEFS	2.928	0.00171	0.0125
3	RHSOFS: GAFS	0.239	0.40553	0.016667
4	RHSOFS: PSOFS	2.002	0.02264	0.025
5	RHSOFS: ACOFS	2.779	0.00273	0.05
